# Supplementary material for: Identification of photoperiod-induced specific miRNAs in the adrenal glands of Sunite sheep (Ovis aries)
Source: Front Vet Sci. 2022 Jul 22;9:888207. doi: 10.3389/fvets.2022.888207 (PMC9354845; doi:10.3389/fvets.2022.888207)
Supplement: Supplementary file 1 [file Data_Sheet_1.ZIP › Supplementary file/miRDeep2 file.zip!/miRDeep2 file/Novel_Structure/SPLP42A3/Novel_148.pdf]

Provisional ID	:	NC_019459.2_5722
Score total	:	2
Score for star read(s)	:	-1.3
Score for read counts	:	0
Score for mfe	:	1.7
Score for randfold	:	1.6
Score for cons. seed	:	
Total read count	:	437
Mature read count	:	437
Loop read count	:	0
Star read count	:	0

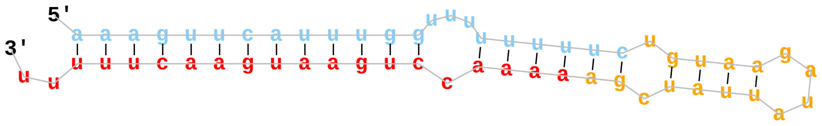

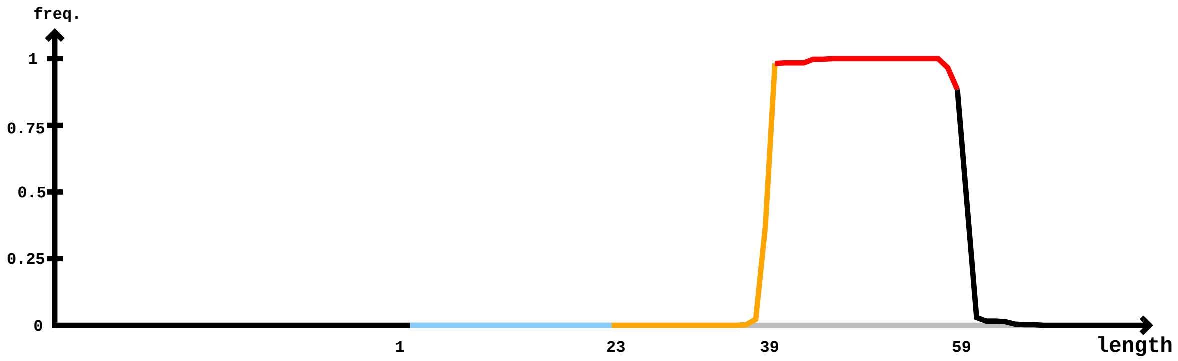


```
                                    Star                                 Mature
5'- cuuaugaaucgucuuucuauuaucagguuggccgaaaaguucauuugguuuuuuuucuguaagauauuaucgaaaaaccugaaugaacuuuuuggccaacucaacccaauac   -3'    exp
    .....(((......)))........(((((((((((((((((((((((...((((((.((((....)))).)))))).)))))))))))))))))))))))...........    reads    mm    sample
    ......................................................................cgaaaaaccugaaugaacuuuu....................    1    0    seq
    .......................................................................gaaaaaccCgaaugaacuuu.....................    1    1    seq
    .......................................................................gaaaaaccAgaaugaacuuuu....................    1    1    seq
    .......................................................................gaaaaaccugaaugaacuuuu....................    4    0    seq
    .......................................................................gaaaaaccCgaaugaacuuuu....................    1    1    seq
    .......................................................................gGaaaaccugaaugaacuuuuu...................    1    1    seq
    .......................................................................gaaaaaccugaaCgaacuuuuu...................    1    1    seq
    ........................................................................aaaaaccugaaugaacCuu.....................    1    1    seq
    ........................................................................aaaaaccCgaaugaacuuu.....................    1    1    seq
    ........................................................................aaaaaUcugaaugaacuuu.....................    12    1    seq
    ........................................................................aaaaaUcugaaugaacuuuu....................    26    1    seq
    ........................................................................aaaaaccugaaGgaacuuuu....................    1    1    seq
    ........................................................................aaaaaccugaaugaacCuuuu...................    1    1    seq
    ........................................................................Gaaaaccugaaugaacuuuuu...................    1    1    seq
    ........................................................................aaaaaUcugaaugaacuuuuu...................    78    1    seq
    ........................................................................aaaaaUcugaaugaacuuuuug..................    24    1    seq
    ........................................................................aaaaaccCgaaugaacuuuuug..................    2    1    seq
    ........................................................................aaaaaccugaaugaacCuuuug..................    1    1    seq
    ........................................................................aaaaaccugaaCgaacuuuuug..................    3    1    seq
    ........................................................................aaaaaccugaaugaacuuuuugg.................    2    0    seq
    .........................................................................aaaaccAgaaugaacuuuu....................    1    1    seq
    .........................................................................aaaaccugaaugGacuuuu....................    1    1    seq
    .........................................................................aaaCccugaaugaacuuuuu...................    1    1    seq
    .........................................................................aaaaccAgaaugaacuuuuu...................    2    1    seq
    .........................................................................aaaaUcugaaugaacuuuuu...................    247    1    seq
    .........................................................................aaaaccugaaugaacuuuuu...................    8    0    seq
    .........................................................................aaaCccugaaugaacuuuuug..................    2    1    seq
    .........................................................................aaaaccugaaugaacuuuuuU..................    2    1    seq
    .........................................................................Gaaaccugaaugaacuuuuug..................    1    1    seq
    .........................................................................aaaaccGgaaugaacuuuuugg.................    1    1    seq
    .........................................................................Gaaaccugaaugaacuuuuugg.................    2    1    seq
    .........................................................................aaaaccugGaugaacuuuuugg.................    1    1    seq
    ..........................................................................aaaccAgaaugaacuuuuug..................    1    1    seq
    .............................................................................ccugaaugaacuuuuuggcc...............    1    0    seq
```

Star Mature

cuuaugaaucgucuuucuauuaucagguuggccgaaaaguucauuugguuuuuuuucuguaagauauuaucgaaaaaccugaaugaacuuuuuggccaacucaacccaauac			
....ccugaaugaacuuuuuggcca..............	4	0	seq
....ccugaaugaacuuuuuggccaa.............	1	0	seq
....ugaGugaacuuuuuggccaacu...........	1	1	seq
